# Applicability of fetal heart rate variation, umbilical artery resistivity index and maternal body temperature for predicting imminent parturition in bitches

**DOI:** 10.1590/1984-3143-AR2025-0103

**Published:** 2026-05-25

**Authors:** Diego Rodrigues Gomes, Diana Villa Verde Salazar, Denise Jaques Ramos, Camila Silveira Stanquini, Tamiris Disselli, Luiz Paulo Nogueira Aires, Stéfany Tagliatela Tinto, Mariana Ambroso Adib Donato Henriques, Alois Foltran Müller, Ricardo Andres Ramirez Uscategui, Adriano Bonfim Carregaro, Marcus Antônio Rossi Feliciano

**Affiliations:** 1 Departamento de Medicina Veterinária, Faculdade de Zootecnia e Engenharia de Alimentos – FZEA, Universidade de São Paulo – USP, Pirassununga, SP, Brasil; 2 Faculdade de Ciências Agrárias e Veterinárias – FCAV, Universidade Estadual Paulista “Júlio de Mesquita Filho” – Unesp, Jaboticabal, SP, Brasil; 3 Facultad de Medicina Veterinaria y Zootecnia, Universidad del Tolima, Ibagué, Tolima, Colombia

**Keywords:** pregnancy, doppler, hemodynamics, delivery prediction, temperature

## Abstract

This study aimed to evaluate the applicability of fetal heart rate variability (FHRvar%), umbilical artery resistivity index (RI-UmbArt), and maternal body temperature (Temp, °C) for predicting parturition in bitches. Fifteen bitches (1-6 years old) were included. Gestational age was estimated using fetal biometry (inner chorionic cavity or biparietal diameter), and during the last week of pregnancy the animals were evaluated twice daily until parturition. Data were retrospectively grouped according to the hours before parturition (HBP). The evaluated parameters were FHRvar% (measured over 5-10 minutes using pulsed Doppler abdominal ultrasonography), RI-UmbArt (assessed by triplex Doppler in three fetuses per session), and Temp (rectal thermometry). Data were statistically compared and correlated with HBP. FHRvar% showed a gradual increase (P = 0.010) beginning at 84 HBP, reaching a peak between 24 and 12 HBP, whereas Temp significantly decreased during the last 24 HBP (P < 0.001). Both FHRvar% and Temp were significantly correlated with HBP (P < 0.001); however, these correlations were weak (Pearson’s r = −0.338 and −0.491, respectively). RI-UmbArt showed no significant variation across HBP (P = 0.711). An FHRvar% > 31.5% predicted parturition within 24 hours with a sensitivity of 65% and a specificity of 67%, whereas a Temp < 37.5°C predicted parturition with 69.6% sensitivity and 77.8% specificity. In conclusion, although FHRvar% and maternal body temperature are influenced by the proximity of parturition, they may not be reliable predictors of parturition timing, while RI-UmbArt appears to remain unchanged close to delivery.

## Introduction

Estimating the expected date of parturition in bitches based solely on breeding dates is unreliable due to variability in the estrous cycle, mating receptivity, and sperm viability within the female reproductive tract (Concannon et al., 1983; Lopate, 2008). Consequently, ultrasound-based prediction methods, including biometric measurements, assessment of organogenesis, and fetal heart rate (FHR) monitoring, have been explored to improve accuracy, with the aim of minimizing gestational losses, particularly in high-value litters and in cases predisposed to dystocia (Miranda and Domingues, 2010; Beccaglia and Luvoni, 2012; Socha et al., 2012; Gil et al., 2014; Beccaglia et al., 2016). Biometric formulas, such as measurement of the inner chorionic cavity (ICC) during early gestation and biparietal diameter (BPD) in later stages, have demonstrated variable accuracy in predicting parturition, with precision decreasing as gestation progresses (Beccaglia and Luvoni, 2012). Moreover, fetal growth rates differ among breeds, with small breeds exhibiting slower growth than large breeds, necessitating adjustments in prediction models (+1 day for small breeds and −2 days for large breeds) (Kutzler et al., 2003; Siena and Milani, 2021).

FHR acceleration and deceleration patterns, as well as the umbilical artery resistivity index (RI-UmbArt), have been investigated as potential indicators of impending parturition in dogs (Gil et al., 2014; Giannico et al., 2016). Previous studies have suggested that increased FHR variability (>30.67%) and decreased RI-UmbArt (<0.7) may predict parturition within 12 hours (Giannico et al., 2016). However, these findings are largely based on methodologies extrapolated from human obstetrics, in which the predictive accuracy of such parameters remains controversial, even when advanced monitoring tools such as cardiotocography are used (Ayres‐de‐Campos et al., 2015; Giannico et al., 2016; Liu et al., 2023). The application of these methods in canine obstetrics is particularly debatable because bitches, unlike ewes, do not share gestational physiology closely resembling that of humans and instead exhibit distinct obstetric characteristics (Figueroa et al., 2011; Bianco et al., 2013; Cui et al., 2023). Additionally, breed variability, gestational age, and individual maternal-fetal factors may further affect the reliability of these assessments (Gil et al., 2014; Giannico et al., 2015).

Assessment of FHR patterns has been investigated in several domestic species, including cattle, horses, and sheep, primarily as a tool for monitoring fetal well-being rather than for predicting the timing of parturition (Jonker et al., 1994; Breukelman et al., 2006; Baska-Vincze et al., 2015; Del’Aguila-Silva et al., 2023). In equine fetuses, no significant FHR variations have been reported during the final days preceding birth (Nagel et al., 2010). In sheep, a decrease in FHR and vaginal temperature during the last week of gestation has been associated with impending parturition (Del’Aguila-Silva et al., 2023). In cattle, some studies have documented FHR decelerations during the last two weeks of gestation (Jonker et al., 1994), whereas others have reported no significant changes (Breukelman et al., 2006). These interspecies discrepancies highlight the need for further investigation into the physiological mechanisms governing FHR changes in bitches.

Given the inconsistencies in the veterinary literature and the limitations of extrapolating human obstetric methodologies to canine pregnancies, we hypothesize that FHR variability (FHRvar%) and RI-UmbArt may not be reliable predictors of parturition. Therefore, the aim of this study was to evaluate their effectiveness as predictors of parturition timing in the bitch.

## Methods

### Ethical statements and animal selection

This study was approved by the Research Ethics Committee of the *Faculdade de Zootecnia e Engenharia de Alimentos (CEUA–FZEA), Universidade de São Paulo*, under protocol CEUA no. 8850100523. Sample size was calculated using G*Power software (version 3.1.9.2; Universität Düsseldorf, Düsseldorf, Germany), based on results from a previous study evaluating the resistivity index (RI) of maternal and fetal vessels in bitches during normal gestation (Di Salvo et al., 2006). A significance level of 5% (α = 0.05) and a statistical power of 70% (1 − β = 0.70) were assumed. The calculation indicated that a sample size of 15 bitches (n = 15) would allow detection of a minimum RI difference of 0.15, exceeding the difference reported as significant in the reference study.

Based on these criteria, 15 healthy pregnant bitches (3 primiparous and 12 multiparous), aged 1–6 years, were included. The animals belonged to the following breeds: one American Bully (nt = 2 puppies); one Weimaraner (nt = 1 puppy); one Yorkshire Terrier with two pregnancies (n_1_ = 7 and n_2_ = 5; nt = 12 puppies); six German Spitz with six pregnancies (n_1_ = 2, n_2_ = 1, n_3_ = 2, n_4_ = 4, n_5_ = 3, and n_6_ = 5; nt = 17 puppies); one Pit Bull (nt = 8 puppies); and four Maltese with four pregnancies (n_1_ = 2, n_2_ = 4, n_3_ = 4, and n_4_ = 4; nt = 14 puppies). Neonatal mortality from birth to 48 h postpartum was 7.4% (n = 4 puppies). One Yorkshire Terrier bitch was included twice, representing two independent pregnancies (Preg. 1 and Preg. 2). A summary of pregnancy data is presented in Table 1.

**Table 1:** Gestational characteristics of 14 bitches included in the study (15 litters) with breed and status distribution Individual data of the 15 bitches included in the study.

**Bitch**	**Breed**	**Pregnancy status**	**Age (years)**	**Puppies per pregnancy**
1	American Bully	Multiparous	6	2
2	Weimaraner*	Singleton	3	1
3	Yorkshire Terrier (preg.1)	Multiparous	3	7
4	Yorkshire Terrier (preg.2)	Multiparous	4	5
5	German Spitz*	Multiparous	3.5	5
6	German Spitz	Singleton	6.5	1
7	German Spitz	Multiparous	3	2
8	German Spitz	Multiparous	6	2
9	German Spitz	Multiparous	6	4
10	German Spitz	Multiparous	6	3
11	Pitbull*	Multiparous	1	8
12	Maltese	Multiparous	6	4
13	Maltese	Multiparous	6	4
14	Maltese	Multiparous	3	4
15	Maltese	Multiparous	5	2

Totals / Statistics: Singleton: 2 (13.33%) / Multiparous: 13 (86.66%); Age range: 1–6.5 years (mean 4.53); Total pregnancies: 15; Live puppies: 54; Stillbirths: 4 (7.4%). *Bitches primiparous. Note: The Yorkshire Terrier appears twice to represent two separate pregnancies. Stillbirth rate refers to deaths from birth to 48 h postpartum.

Exclusion criteria included a history of cardiovascular, hepatic, renal, or endocrine disease, hypertension, or inadequate vaccination or parasite control. Written informed consent was obtained from all owners prior to inclusion in the study.

### Pregnancy confirmation and gestational age estimation

Owners were instructed to record mating or insemination dates and to present their bitches for evaluation approximately 30 days later (range: 20–55 days). At this initial visit, pregnancy was confirmed by abdominal ultrasonography, and embryonic or fetal age was estimated as days before parturition (DBP).

Before 35 days of gestation, the presence of gestational vesicles confirmed pregnancy, and DBP was estimated using the following formulas: [DBP = (ICC in mm − 68.68) / 1.53] for small breeds and [DBP = (ICC in mm − 82.13) / 1.80] for medium breeds.

After 35 days of gestation, DBP was estimated using: [DBP = (BPD in mm − 25.11) / 0.61] for small breeds and [DBP = (BPD in mm − 29.18) / 0.70] for medium breeds (Beccaglia and Luvoni, 2006, 2012).

Mating information, organogenesis assessment, and estimated DBP were used to determine the onset of monitoring, which began approximately 7 days before the expected date of parturition.

### Ultrasound examination techniques

All ultrasound examinations were performed using a MyLab™ X8VET ultrasound system (Esaote, Genoa, Italy) equipped with a linear multifrequency transducer (4–15 MHz) and a microconvex transducer (3–11 MHz). The microconvex transducer was primarily used for fetal biometry and deeper structures (medium and large-sized bitches), whereas the linear transducer was used for qualitative evaluation of embryonic and fetal development. For fetal heart rate (FHR) and RI-UmbArt assessments, the microconvex transducer was preferred due to its superior insonation angle.

Bitches were positioned in dorsal or lateral recumbency on a padded examination table to minimize stress. Abdominal hair was clipped, and conductive gel was applied. Scanning began at the xiphoid region and proceeded in a clockwise circular pattern to examine the entire abdominal cavity.

Fetuses were assessed in a standardized order: first in the right uterine horn, then in the left, and finally near the uterine bifurcation. To avoid redundant measurements in large litters, at least three fetuses were evaluated per examination whenever possible: the most cranial fetus in each uterine horn and the fetus closest to the bifurcation. In total, at least 37 fetuses were evaluated. Image settings (gain, focus, and depth) were individually adjusted for each fetus, with a maximum imaging depth of 4 cm (Simões et al., 2018).

### Last week examinations

During the final week of gestation (approximately seven days before the estimated date of parturition), physical, obstetric, and laboratory evaluations were initially performed. Physical examination included rectal temperature measurement, cardiac auscultation, capillary refill time, and lymph node palpation. Laboratory analyses included complete blood count (CBC), serum creatinine, urea, alanine aminotransferase (ALT), alkaline phosphatase (ALP), total protein, albumin, calcium, and glucose concentrations. These assessments aimed to identify clinical or hematological abnormalities and anticipate potential indications for caesarean section.

Serial ultrasound examinations were then initiated and performed twice daily at approximately 12-hour intervals until parturition. The time of parturition was defined as 0 hours before parturition (HBP), and previous examinations were retrospectively grouped into ten intervals: 120–108 h, 108–96 h, 96–84 h, 84–72 h, 72–60 h, 60–48 h, 48–36 h, 36–24 h, 24–12 h, and 12–0 h. The evaluated parameters were fetal heart rate variability (FHRvar%), umbilical artery resistivity index (RI-UmbArt), and maternal rectal temperature (Temp, °C).

For FHRvar% assessment, each fetus was evaluated for 5 minutes using spectral Doppler. The sampling cursor was positioned within the fetal cardiac ventricle, and measurements were obtained from at least three artifact-free spectral waves within a 5-second time window. Minimum and maximum FHR values (bpm) were recorded from a minimum of 10 measurements per fetus at each time point (every 12 h). The FHR gradient was calculated as the difference between maximum and minimum values, and FHRvar% was calculated using the formula:

[FHRvar% = (100 × FHR gradient) / maximum FHR] (1)

For example, in a pregnancy with two fetuses, 20 FHR measurements were obtained at each time point, allowing calculation of FHRvar% per HBP group (Giannico et al., 2016).

**Figure 1:**
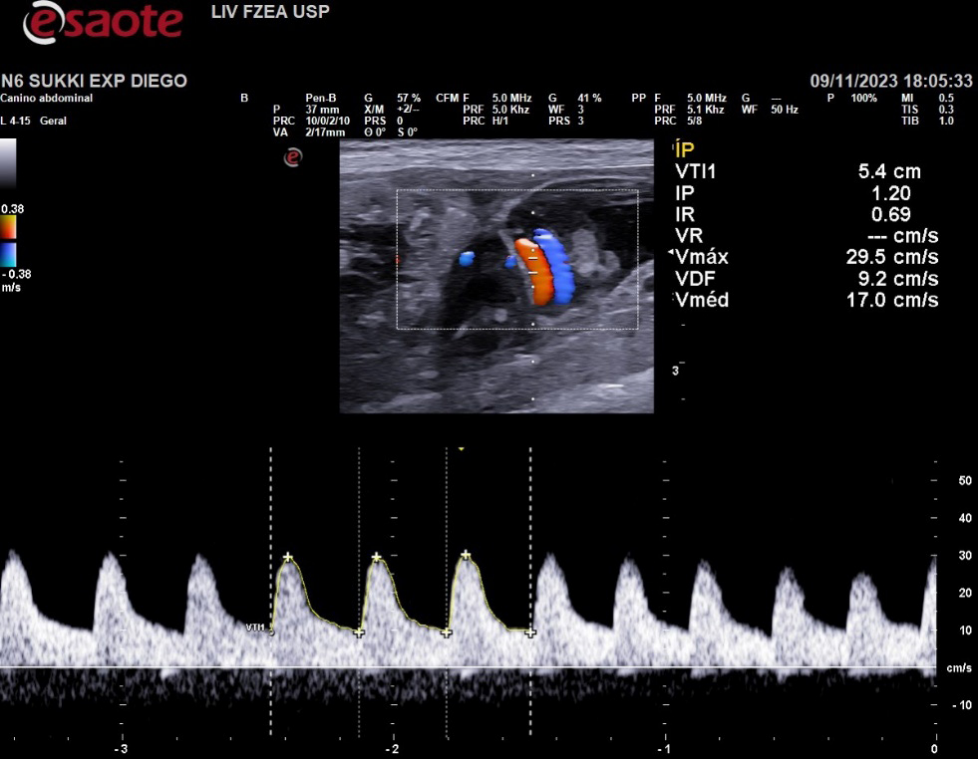
Triplex Doppler ultrasound of the umbilical artery of a naturally delivered canine fetus. The activated color Doppler identifies the umbilical artery (red) and vein (blue). The spectral Doppler, with three identical consecutive waves, graphically demonstrates the systolic peak velocity (SPV) and final diastolic velocity (FDV), resulting in the automatic calculation of the RI.

For RI-UmbArt measurement, the umbilical artery was identified using B-mode ultrasonography and confirmed by color Doppler. Image optimization included adjustment of insonation angle and pulse repetition frequency, maintenance of Doppler gain below 50%, scale and baseline adjustment to fully display the spectral waveform, and placement of a 2–3 mm sampling cursor in the center of the umbilical cord, with an insonation angle <60° to minimize waveform distortion. Three artifact-free waveforms were selected (Figure 1), and the ultrasound system automatically calculated peak systolic velocity, end-diastolic velocity, time-averaged minimum and maximum velocities, pulsatility index, and resistivity index (Gasser et al., 2020). The mean RI value was recorded as RI-UmbArt for each HBP group (Di Salvo et al., 2006; Giannico et al., 2016).

Maternal rectal temperature was measured twice daily (morning and afternoon) using the same digital thermometer and by the same operator. Measurements were initiated when nesting behavior was observed, ensuring proper placement of the thermometer in the rectal ampulla with lateral contact with the rectal wall. Temperature data were standardized and analyzed from 60 HBP to parturition.

In all cases, the decision to perform a caesarean section was based on nesting behavior, a decline in maternal body temperature, and, primarily, evidence of fetal distress (persistent FHR between 150 and 180 bpm for more than 3 minutes), according to Gil et al. (2014) and Smith (2012).

### Statistical analysis

Statistical analyses were performed using GraphPad Prism software (version 8.0.0 for Windows). FHRvar%, RI-UmbArt, and Temp (°C) were compared among HBP groups, type of parturition (natural vs. caesarean section), and litter size (singleton vs. multiple) using the Mann–Whitney or Kruskal–Wallis tests. When significant differences were detected, Dunn’s post hoc test was applied.

Pearson’s correlation analysis was subsequently performed between real (non-grouped) HBP values and FHRvar%, RI-UmbArt, or Temp (°C). When significant correlations were identified, simple linear regression models were constructed using HBP as the dependent variable and the evaluated parameters as predictors, with the aim of developing predictive equations.

Finally, receiver operating characteristic (ROC) curve analysis was performed for variables showing significant differences, estimating optimal cut-off values, sensitivity, specificity, and area under the curve (AUC) for predicting parturition timing.

For all analyses, statistical significance was set at P < 0.05, and results are presented as median ± interquartile range (IQR).

## Results

Of the bitches included in the study, four (4/15) required caesarean section. In two of these cases (2/4), nesting behavior followed by obstructive dystocia was observed. Among the six bitches (6/15) that delivered one or two puppies, only one bitch carrying two puppies required caesarean section due to breed-related factors (American Bully) after the identification of fetal distress.

**Figure 2:**
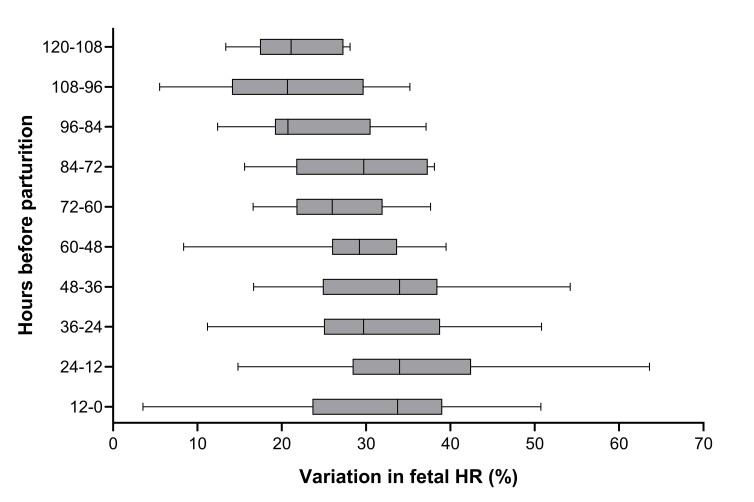
Boxplot graph of percentage variation in fetal heart rate (FHR) at different time intervals before delivery, from -120 hours to the time of parturition (0h). Each boxplot represents the distribution of the percentage variation in FHR in a specific interval, with the central line indicating the median, the boxes delimiting the quartiles and the extension lines representing the minimum and maximum values.

Fetal heart rate (FHR) acceleration and deceleration, expressed as an increase in FHRvar%, began earlier than 120 hours before parturition (HBP) in some bitches. Mean FHRvar% showed a gradual increase (P = 0.010) starting at 84 HBP and reached its highest values between 24 and 12 HBP. However, there was a wide overlap among HBP intervals, indicating substantial individual variability and limiting the accuracy of this parameter as an isolated predictor of parturition timing (Table 2; Figure 2). This increase was independent of the type of parturition (P = 0.440). Nevertheless, FHRvar% was significantly lower in singleton pregnancies (P = 0.046), with a mean variation of 22.9%, compared with multiple pregnancies, which showed a mean variation of 29.2%.

**Table 2:** median ± interquartile range (IQR) of fetal heart rate variation (FHRvar%), umbilical artery resistivity index (IR-UmbArt) and maternal body temperature (Temp °C), in the hours before parturition (HBP). N represents the number of measurements in each time and variable.

**HBP**	**n**	**FHRvar%**	***P* Value**	**n**	**IR-UmbArt**	***P* Value**	**n**	**Temp**	***P* Value**
120-108	6	21.1±9.98^a^	0.010	6	0.76±0.10	0.7114	0	N/A	0.0002
108-96	8	20.7±15.6^a^		8	0.76±0.06		0	N/A	
96-84	9	20.7±11.3^a^		8	0.78±0.06		0	N/A	
84-72	10	29.7±15.6^b^		10	0.79±0.07		0	N/A	
72-60	13	26.0±10.2^b^		12	0.77±0.05		0	N/A	
60-48	14	29.2±7.71^bc^		14	0.74±0.08		13	37.9±0.43^a^	
48-36	13	33.9±13.6^c^		13	0.75±0.07		12	37.8±0.61^ab^	
36-24	15	29.7±13.8^bc^		15	0.75±0.08		15	37.8±0.39^a^	
24-12	19	33.9±14.0^c^		19	0.78±0.08		19	37.6±0.94^b^	
12-0	21	33.7±15.4^c^		15	0.76±0.10		13	37.3±0.37 ^bc^	

Values presented as median ± interquartile range. The medians of the HBPs that do not share a letter are significantly different from each other, according to Dunn's post-test (p < 0.05).

**Figure 3:**
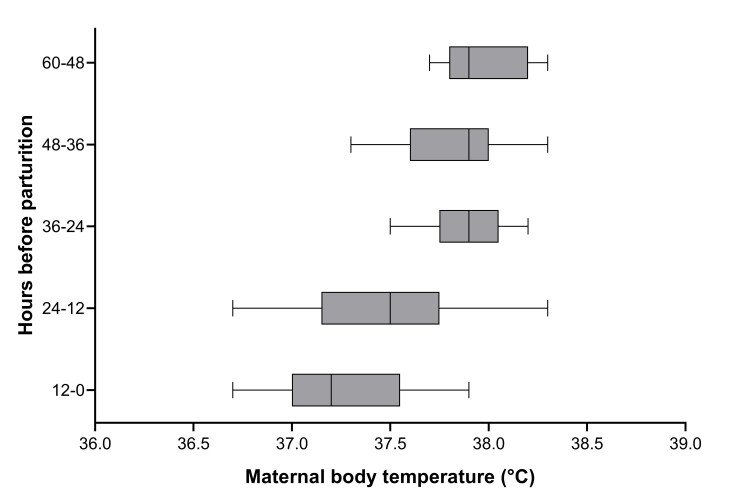
Boxplot graph of maternal body temperature (°C) in pregnant bitches at different time intervals before delivery, from -60 hours to the time of parturition (0h). Each boxplot represents the distribution of the maternal body temperature (Temp) in a specific interval, with the central line indicating the median, the boxes delimiting the quartiles and the extension lines representing the minimum and maximum values.

Maternal body temperature showed a significant decrease as parturition approached (P < 0.001) (Table 2; Figure 3). This parameter was not influenced by the type of parturition (P = 0.246) or by litter size (P = 0.865).

**Figure 4:**
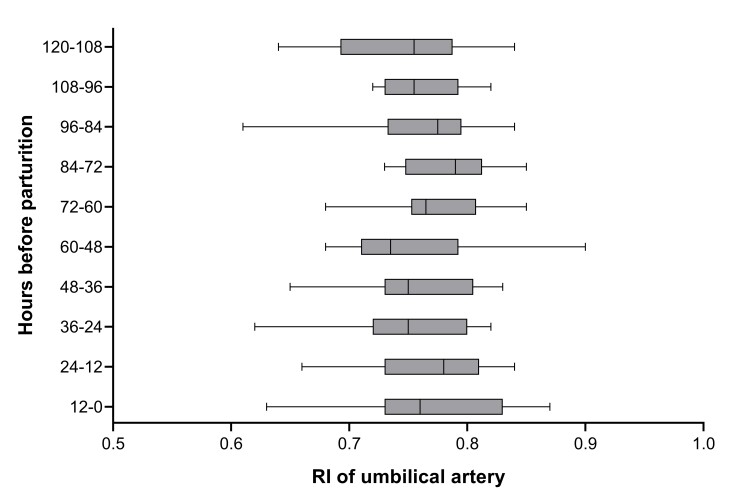
Boxplot graph of the umbilical artery resistivity index in pregnant bitches at different time intervals before delivery, from -120 hours to the time of parturition (0h). Each boxplot represents the distribution of the umbilical artery resistivity index (RI) in a specific interval, with the central line indicating the median, the boxes delimiting the quartiles and the extension lines representing the minimum and maximum values.

The umbilical artery resistivity index (RI-UmbArt) did not show significant variation across HBP intervals (P = 0.711) (Table 2; Figure 4) and was not influenced by the type of parturition (P = 0.099) or litter size (P = 0.431). Furthermore, no significant correlation was observed between RI-UmbArt and HBP (P = 0.828; Pearson’s r = −0.019).

Both FHRvar% and maternal body temperature showed significant negative correlations with HBP (P < 0.001); however, these correlations were weak (Pearson’s r = −0.338 and −0.491, respectively). Although simple linear regression models were statistically significant (P < 0.050) for FHRvar% and Temp as predictors of HBP (HBP = 98.17 − 1.468 × FHRvar% and HBP = −36.31 + 0.9906 × Temp), the coefficients of determination were low (R^2^ = 15.4% and 27.8%, respectively), indicating limited predictive reliability. To further explore the data, a best-subsets regression model including RI-UmbArt, FHRvar%, and Temp were applied. However, none of the proposed combinations substantially improved model performance: FHRvar% + Temp (R^2^ = 29.3%), RI-UmbArt + Temp (R^2^ = 29.3%), RI-UmbArt + FHRvar% (R^2^ = 1.0%), or the combination of all three variables (R^2^ = 28.9%).

**Figure 5:**
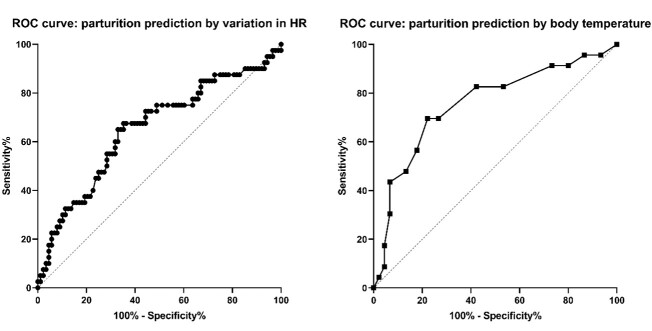
ROC curves of fetal heart rate variation (%, left) and maternal body temperature (°C, right) as predictors of parturition within the next 24 hours.

Receiver operating characteristic (ROC) curve analysis (Figure 5) for parturition prediction showed that an FHRvar% > 31.5% predicted parturition within the subsequent 24 hours (P = 0.006), with a sensitivity of 65% (95% CI 49.5% to 77.9%), a specificity of 67% (95% CI 56.7% to 75.9%), and an area under the curve (AUC) of 65.3%. Similarly, a maternal body temperature < 37.5°C predicted parturition within the next 24 hours (P < 0.001), with a sensitivity of 70% (95% CI 49.13% to 84.4%), a specificity of 78% (95% CI 63.7% to 87.5%), and an AUC of 75.6%. When both predictors were combined (Temp < 37.5°C and FHRvar% > 31.5%), sensitivity decreased to 39.0%, whereas specificity increased to 91.0%, with an AUC of 65%. Although specificity markedly improved, the overall predictive performance was reduced due to the low sensitivity of the combined model.

## Discussion

The gestational age estimation formulas used to guide the onset of last-week pregnancy evaluations are well established in routine clinical practice and demonstrate acceptable accuracy when using both ICC (85.9%) and BPD (95.2%), with low bias (±2 days) (Beccaglia and Luvoni, 2006, 2012). These formulas allowed standardized grouping of examinations according to hours before parturition (HBP).

The predictive accuracy of maternal body temperature (sensitivity 70%, specificity 78%), fetal heart rate variability (FHRvar%; sensitivity 65%, specificity 67%), and umbilical artery resistivity index (RI-UmbArt; no significant predictive value) was limited. Even when Temp and FHRvar% were combined, predictive performance further declined, mainly due to markedly reduced sensitivity (39%) and a low coefficient of determination (R^2^ = 29.3%). Ideally, a reliable predictor of parturition should demonstrate high accuracy (sensitivity and specificity >90%), as false-positive or false-negative predictions may have severe consequences for the maternal–fetal unit, including unnecessary interventions such as caesarean section (Cohen et al., 2021).

The FHRvar% cutoff value >31.5% showed low diagnostic accuracy for predicting parturition within the subsequent 24 hours and was slightly higher than the threshold proposed by Giannico et al. (2016), who reported a high probability of parturition within 12 hours using a cutoff value >30.7%. This discrepancy may be explained by the substantial overlap in FHRvar% values observed during the days preceding delivery. Values suggestive of imminent parturition (≤12 h) were also detected two to four days earlier in the present study. Such overlap should not be overlooked, and serial evaluations are therefore recommended, including assessment of the maximum number of fetuses for at least three minutes, as this approach improves the detection of fetal distress, characterized by sustained FHR values between 150 and 180 bpm, or uterine inertia–associated dystocia, in which FHR values return above normal without oscillation (Gil et al., 2014; Lezama-García et al., 2023).

Careful peripartum fetal evaluation may reduce neonatal mortality, as respiratory distress syndrome and hypoxia account for more than 60% of neonatal deaths within the first 48 hours postpartum, particularly in miniature and small breeds (Münnich and Küchenmeister, 2009). No intervention should be based solely on FHR assessment, given its high physiological variability during the final days of gestation. In the authors’ clinical experience, reliance on FHR variation as the sole criterion for caesarean section may contribute to reduced neonatal survival.

In human fetuses, FHR regulation is primarily mediated by the autonomic nervous system, with sympathetic dominance in early gestation and increasing parasympathetic influence near parturition, particularly in response to hypoxia-induced decelerations (Tournier et al., 2022). In veterinary medicine, most research in this field has focused on sheep, due to similarities with human maternal-fetal physiology and their frequent use as experimental models. In sheep, fetal cardiovascular responses to hypoxia and acidemia have been associated with adverse neonatal outcomes (Rivolta et al., 2014; Morrison et al., 2018).

In canine pregnancies, FHR assessment is widely accepted for identifying fetal distress; however, knowledge of normal and abnormal fetal physiology during the peripartum period remains limited, particularly regarding the relationship between uterine contractions and FHR acceleration and deceleration patterns (Zone and Wanke, 2001; Miranda and Domingues, 2010; Gil et al., 2014; Lezama-García et al., 2023). In the present study, transient FHR acceleration and deceleration were observed as early as 120 HBP during the last week of pregnancy. This finding highlights the risk of misdiagnosing fetal distress or imminent labor when evaluations rely exclusively on FHR, without considering prodromal signs, maternal temperature, or serum progesterone concentrations (Concannon and Rendano, 1983; Socha et al., 2012; Grellet et al., 2018; Lopate, 2023). Our results demonstrated that FHR variations exceeding 30% were not exclusive to the final 12 HBP, in contrast to previous descriptions by Giannico et al. (2016).

It has been hypothesized that increased FHR variability may result from subtle uterine contractions preceding parturition (Gil et al., 2014). Based on our findings, we further hypothesize that these preparatory contractions in bitches may resemble Braxton–Hicks contractions described in the third trimester of human pregnancy, which are sporadic contractions that may be imperceptible or painful and may begin several days before labor (La Verde et al., 2022). In bitches, such contractions may occur as early as five days before parturition. Further studies using prolonged cardiotocographic monitoring are warranted to test this hypothesis.

Lower FHR variability was observed in singleton pregnancies that progressed to natural parturition, further complicating the identification of both imminent delivery and fetal distress in these cases. Parturition signaling in singleton or small litters is believed to be associated with delayed or incomplete luteolysis, influenced by fetal number, which may postpone or even prevent spontaneous labor onset. Cortes et al. (2025) reported that pregnancies with one or two puppies accounted for more than 70% of cases without progesterone decline on the day of caesarean section compared with pregnancies carrying three or more fetuses. Although Blanco et al. (2020) and Cortes et al. (2025) did not observe differences in mean FHR among bitches of different body weights or litter sizes, the latter study reported a positive correlation between minimum FHR on the day of caesarean section and neonatal survival, reinforcing the importance of early detection of fetal distress.

Maternal body temperature showed a significant decline before parturition, corroborating previous reports (Concannon, 2000; Geiser et al., 2014; Grellet et al., 2018; Tsutsui and Murata, 1982). This decrease was evident up to 60 hours before parturition and proved more accurate than FHRvar% and RI-UmbArt for estimating delivery timing. The temperature drop is directly associated with a sudden decline in progesterone, a thermogenic hormone acting on thermosensitive neurons (Nakayama et al., 1975; Grellet et al., 2018; Karnezi et al., 2020). Body temperature typically returns to baseline at parturition, emphasizing the importance of serial measurements during the final gestational week (Concannon and Hansel, 1977; Grellet et al., 2018). Although more reliable than the other evaluated variables, maternal temperature alone remains insufficient for precise prediction and should be interpreted cautiously, particularly given metabolic differences among breeds of varying sizes. Baseline temperature should be established for each individual through repeated daily measurements during the last week of gestation. A temperature decrease of 1.1–1.7°C appears to be a useful indicator, as small breeds may reach temperatures as low as 35°C within 18–6 hours before parturition, whereas medium-sized breeds rarely fall below 36°C and large breeds may remain close to 37°C (Johnson, 2008; Grellet et al., 2018).

Canine gestation is relatively short, and fetal organ maturation continues into the postnatal period (Lopate, 2008; Vannucchi et al., 2012). This is particularly relevant when planning elective caesarean sections, which should only be performed after clear signs of parturition. The physiological stress associated with spontaneous labor promotes extrauterine respiratory adaptation and reduces stillbirth rates. Moreover, surfactant production is insufficient in fetuses delivered before 62 days after the LH peak (Kutzler, 2008). As an altricial species, canine neonatal survival is highly dependent on maternal care and adequate fetal maturation, since the transition from intrauterine to extrauterine life is abrupt and pulmonary development remains functionally and morphologically immature even after birth (Schittny, 2017; Teifke et al., 2023).

Few veterinary studies have evaluated neonatal mortality beyond the immediate neonatal period up to weaning (Grundy, 2023). In humans, increased infant mortality has been associated with caesarean delivery, suggesting that reduced mortality is linked to clinically indicated rather than elective procedures (Paixão et al., 2021). Based on our findings, FHRvar% and RI-UmbArt, as in human obstetrics, should primarily be used to detect or monitor fetal distress rather than to predict parturition. A multivariate peripartum evaluation integrating gestational age estimation formulas, serial progesterone measurements, maternal body temperature, and nesting behavior is likely to support more accurate clinical decision-making (Münnich and Küchenmeister, 2009; Wydooghe et al., 2013; De Cramer and Nöthling, 2018). Given their limited predictive value when used in isolation, FHRvar%, RI-UmbArt, and maternal temperature should not be relied upon solely for scheduling caesarean sections.

Miranda and Domingues (2010) reported that RI-UmbArt decreases with advancing gestation, as observed in humans, and plays a role in the appearance of diastolic flow approximately 21 days before parturition. This decrease is associated with increased perfusion required for fetal growth, placental maturation, and fetal cardiovascular development. In contrast, the quantitative assessment of RI-UmbArt in the present study demonstrated stability during the final gestational week, and the spectral waveform pattern, characteristic of an intermediate resistance vascular bed with decreasing diastolic flow, was similar to that described by Freitas et al. (2016), who found no differences between normal and abnormal pregnancies. Although Freitas et al. (2016) reported increased RI values (>0.8) in cases of fetal underdevelopment, our study identified no obvious adverse neonatal outcomes in fetuses with RI-UmbArt values exceeding 0.8 at term. Mean RI-UmbArt values during the last week of gestation were consistent with those previously reported (Di Salvo et al., 2006; Freitas et al., 2016), supporting the conclusion that RI-UmbArt remains relatively stable close to parturition.

Evaluation of the umbilical artery in the free portion of the umbilical cord, although common in both human and veterinary practice (Giannico et al., 2016; Khare et al., 2006), may not be optimal, as Doppler-derived indices can vary by up to 29–46% depending on sampling location in normal human pregnancies (Maulik et al., 1989). Doppler indices tend to be higher near the fetal abdominal insertion and lower near placental insertion (Maulik et al., 1989; Khare et al., 2006), which may explain the individual variability observed in the present study. Given the spatial constraints imposed by multiple fetuses and limited intrauterine space near term, reproducibility of free-cord measurements in bitches may be limited. Future studies should consider standardizing RI-UmbArt measurements at fixed, easily identifiable locations, such as the intraplacental insertion site, fetal abdominal insertion site, or perivesical intra-abdominal segment.

Body weight and breed size have also been shown to influence RI values. Blanco et al. (2020) reported lower uterine artery RI values in large compared with small bitches, attributing this difference to greater maternal–fetal vascularization associated with larger litters and heavier fetuses. Similar findings have been reported by Di Salvo et al. (2006), Giannico et al. (2015), and Freitas et al. (2016), who observed lower RI values in larger breeds regardless of litter size. In the present study, mean RI-UmbArt values were comparable to those reported by Blanco et al. (2020), who also evaluated predominantly small breeds.

Several limitations of this study should be acknowledged. Primiparous and multiparous bitches, as well as singleton and multiple pregnancies, were analyzed together due to the limited number of cases, which restricted statistical power. Physiological differences related to parity and litter size should therefore be considered when interpreting these findings (Eilts et al., 2005; Cortes et al., 2025). Additionally, the absence of precise gestational timing markers, such as progesterone measurement at ovulation and at parturition, cytological assessment of diestrus onset, and inter-operator variability, represents a limitation. Although the study was not blinded, ultrasound data were analyzed only after completion of data collection. Fetal distress detected by FHR evaluation was the sole ultrasonographic criterion used to guide intervention, in conjunction with clinical findings.

## Conclusions

Under the conditions of this study, fetal heart rate acceleration and deceleration, umbilical artery resistivity index, and maternal rectal temperature were not accurate predictors of parturition timing and showed only weak correlations with hours before parturition. Among the evaluated variables, maternal body temperature demonstrated the highest predictive value; however, it remains insufficient when used as a standalone parameter. Notably, significant FHR variability was observed as early as 120 hours before parturition, suggesting that decision-making based solely on this parameter may increase the risk of premature intervention. Finally, RI-UmbArt values recorded during the final week of canine gestation were consistent with those reported for normal pregnancies and did not correlate with the timing of parturition.

## Data Availability

Research data is only available upon request.
